# Potential Utility of Natural Products against Oxidative Stress in Animal Models of Multiple Sclerosis

**DOI:** 10.3390/antiox11081495

**Published:** 2022-07-29

**Authors:** Zheng Zha, Sisi Liu, Yijiang Liu, Chen Li, Lei Wang

**Affiliations:** Beijing Key Lab of TCM Collateral Disease Theory Research, School of Traditional Chinese Medicine, Capital Medical University, Beijing 100069, China; zhazheng@ccmu.edu.cn (Z.Z.); liusisi@mail.ccmu.edu.cn (S.L.); liuyijiang@ccmu.edu.cn (Y.L.); lichen@mail.ccmu.edu.cn (C.L.)

**Keywords:** multiple sclerosis, animal model, oxidative stress, natural products, antioxidants

## Abstract

Multiple sclerosis (MS) is an autoimmune-mediated degenerative disease of the central nervous system (CNS) characterized by immune cell infiltration, demyelination and axonal injury. Oxidative stress-induced inflammatory response, especially the destructive effect of immune cell-derived free radicals on neurons and oligodendrocytes, is crucial in the onset and progression of MS. Therefore, targeting oxidative stress-related processes may be a promising preventive and therapeutic strategy for MS. Animal models, especially rodent models, can be used to explore the in vivo molecular mechanisms of MS considering their similarity to the pathological processes and clinical signs of MS in humans and the significant oxidative damage observed within their CNS. Consequently, these models have been used widely in pre-clinical studies of oxidative stress in MS. To date, many natural products have been shown to exert antioxidant effects to attenuate the CNS damage in animal models of MS. This review summarized several common rodent models of MS and their association with oxidative stress. In addition, this review provides a comprehensive and concise overview of previously reported natural antioxidant products in inhibiting the progression of MS.

## 1. Introduction

Multiple sclerosis (MS) is a classical autoimmune disease of the central nervous system (CNS) [1] that occurs mainly in young adults, with a prevalence rate of approximately 2 to 150 cases per 100,000 people [2]. Currently, there are approximately 2.8 million people worldwide with MS [3], with a male to female ratio of 1:2–1:3 [4]. The majority of patients initially present with the relapsing-remitting type of the disease, which evolves to a secondary progressive type within 5–15 years [5]. MS is associated with a high disability rate and has a significant negative impact on the health and quality of life of patients. The aetiology and pathogenesis of MS are unclear and may be associated with genetic, environmental, viral and immune factors [6]. The main pathological features of MS include inflammatory response, demyelination and axonal and neuronal injury [7]. Immunoinflammation-induced demyelination is the main cause of severe neurological dysfunction in patients with MS. Due to the spatial and temporal multiplicity of the lesions and the disease course [8], respectively, the clinical symptoms of MS are complex and recurrent. The primary symptoms of MS include blurred vision, motor abnormalities such as limb weakness and paralysis, sensory abnormalities such as numbness, tingling sensation and zonesthesia, as well as urinary and bowel disorders and memory loss that worsen progressively [9,10]. Inadequate remyelination at the site of injury results in neurodegenerative lesions leading to chronic disability [11]. Furthermore, 80% of the patients experience a relapsing-remitting course, eventually progressing to paralysis or blindness [12]. The prevalence of MS has been increasing worldwide over the past decades; however, there are still some limitations that restrain the efficacy and safety of MS treatment. Glucocorticoids, interferons and immunomodulators are the main modern medical agents that were used in the treatment of MS [13]; however, the associated adverse effects hinder their large-scale application [14]. Despite the indefinite aetiology, the pathological characteristics of MS have been clearly described and chiefly comprise inflammatory cell (peripheral origin) infiltration, oligodendrocyte damage, destruction of neuronal axons and activation of resident immune cells (RICs) in the CNS [15]. Such changes have been observed within the white and grey matter regions of the brain and the spinal cord. Moreover, recent studies have reported that the rapid response of the RICs within the CNS, especially the activation of microglia [16] and astrocytes [17], is associated with the expression and release of oxidative stress-related molecules [18,19], which have a significant effect on chronic myelin loss and impede remyelination and repair [20].

**Figure 1:**
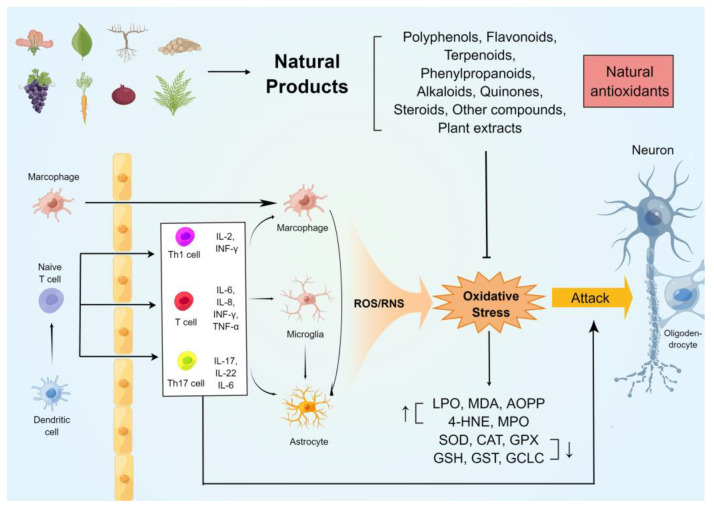
The neuroprotective effects of natural products in animal models of MS. Natural products alleviated ROS/RNS overgeneration, lipid peroxidation, oligodendrocytes loss, and neuronal injury; increased antioxidant enzymes, lipid metabolism, and myelin repair, among others.

The oxidative stress on the immune and neuronal cells in MS was generalizedin this review. In addition, several commonly used rodent models of MS were studied, and their relationship with oxidative damage was summarized. Furthermore, we highlighted the potential antioxidant and anti-demyelinating effects of natural compounds of plant origin. Figure 1 summarises the mechanisms of oxidative stress generation in the CNS of animal models of MS and the neuroprotective effects of natural antioxidants.

## 2. Oxidative Stress in the CNS of Patients with MS

Oxidative stress plays a crucial role in inflammatory demyelinating diseases. In the CNS of patients with MS, oxidative stress interferes with proteins, lipids and DNA [21] in the neurons and glial cells to promote oxidative degradation and subsequent loss of function. In addition, oxidative stress induces mitochondrial damage, leading to the collapse of the mitochondrial membrane potential and a further increase in the production of reactive oxygen species (ROS) and reactive nitrogen species (RNS), thereby exacerbating demyelination and neurodegeneration [22]. It has been confirmed that patients with MS have elevated levels of oxidative stress markers such as total oxidative status (TOS), malondialdehyde (MDA), and oxidative stress index (OSI) while impaired expression of antioxidant markers, including total antioxidant status (TAS) and superoxide dismutase (SOD) [23]. However, understanding the relationship between MS’s pathogenesis and oxidative stress needs to be further improved. It has been reported that an initial stimulus for MS produces low concentrations of damage-associated molecular patterns (DAMPs) in CNS tissue, which participate in an immune response to repair injured tissues. Moreover, the relapsing stage of this disease is characterized by a highly destructive tissue environment, where increased levels of DAMPs are produced during acute inflammation, leading to a chain reaction of damage. High rates of proinflammatory DAMPs could promote an extensive tissue injury, amplifying the DAMPs levels in tissue significantly locally and systemically, which could create a perpetual state of tissue injury. It has been proposed that suppressing DAMPs to avoid the activation of Toll-like receptors (TLRs) could be considered a new potential target in treating autoimmune diseases such as MS, which may offer feasible alternatives to improve current therapies [24]. It is well known that TLRs are a class of membrane proteins, which are widely expressed on the surface of many immune cells and play an essential role in causing the activation of microglia and astrocytes into a proinflammatory state, thus can effectively mediate neuroinflammation [25].

Activated microglia and astrocytes are often considered the main source of ROS and nitric oxide (NO) in the CNS [26]. Extensive oxidative damage to lipids, proteins and nucleic acids is observed in the active demyelinated regions [27], particularly around reactive astrocytes and myelin debris-rich macrophages [28]. The damage induced by astrocytes is mainly caused by oxidative stress mediated by nicotinamide-adenine dinucleotide phosphate (NADPH) and mitochondrial dysfunction [29]. NADPH oxidase, a multisubunit enzyme complex, is activated and catalyzes the production of superoxide from oxygen under pathological conditions within the astrocytes [30,31]. Reactive astrocytes release inflammatory cytokines and ROS and form glial scars that impede axonal growth and regeneration [32]. In addition, inducible nitric oxide synthase (iNOS) has been detected in astrocytes within the active demyelinating foci, suggesting that astrocyte-derived NO produced via iNOS may contribute to oxidative stress-mediated neurotoxicity [33,34]. NO released from reactive astrocytes can increase mitochondrial damage and induce DNA strand breaks leading to neuronal death [35,36]. In MS, activated microglia produce a variety of inflammatory mediators, including NO and superoxide [37,38], to form peroxynitrite (ONOO^−^), eventually inducing oxidative stress-mediated neuronal damage [39]. Compared to astrocytes, iNOS in the hippocampal microglia are more sensitive to lipopolysaccharide stimulation [40,41]. In addition, arginine (Arg) is an important substrate for nitric oxide synthase (NOS). An insufficient amount of arginine in the microglia can lead to iNOS-mediated NO and superoxide production [42,43], resulting in the formation of the more toxic ONOO^−^.

During the progression of MS, T lymphocytes invade the CNS and attack the myelin sheath resulting in rapid activation of intrinsic immune cells in the brain [44,45]. The high expression and continuous activation of NADPH oxidase (NOX), myeloperoxidase, cyclooxygenase and iNOS in the activated microglia [46] lead to the generation of a large number of abnormal free radicals, including superoxide anion (O_2_•^−^), hydrogen peroxide (H_2_O_2_), hydroxyl (•OH), ONOO^−^ and nitric dioxide (NO_2_•) [47]. Free radicals are highly reactive molecules with unpaired electrons in their outer orbits. These molecules can extract electrons from other biomolecules to generate new free radicals, triggering a chain reaction and ultimately causing extensive cellular damage [48].

Free radicals can damage many types of nerve cells and have the strongest effect on oligodendrocytes and neurons [49]. In addition, the sites with the most extensive oxidative damage in MS are observed in the myelin sheath and oligodendrocytes due to their weak antioxidant defense systems, high endogenous metabolic activity rates and low intrinsic antioxidant enzyme levels [50]. The enzymatic and non-enzymatic antioxidant defence activities are limited in oligodendrocytes [51], where superoxide dismutase (SOD) is essential for the enzymatic conversion of O_2_•^−^ to H_2_O_2_ and O_2_. H_2_O_2_ is decomposed by catalase (CAT) and converted to H_2_O and O_2_. Glutathione peroxidase (GPx) promotes the conversion of H_2_O_2_ to reduced glutathione (GSH) to produce H_2_O and oxidised glutathione (GSSG) [52,53]. All of these antioxidant enzymes are minimally expressed in oligodendrocytes [54]. In addition, the iron content in oligodendrocytes is high due to the involvement of iron in differentiation and myelination [55]. However, as a catalyst for the Fenton reaction, iron converts H_2_O_2_ to the highly reactive •OH [56]. Immature oligodendrocytes are more sensitive to oxidative stress and free radical damage than mature oligodendrocytes due to the higher concentration of unsaturated fatty acids within oligodendrocyte precursor cells (OPC), as well as an increase in mitochondrial energy and oxygen consumption rate during differentiation [57]. In MS, free radicals represented by ONOO^−^ significantly reduced the mitochondrial respiration and membrane potential in oligodendrocytes, leading to DNA strand break-mediated cell death [58]. Mitochondrial dysfunction leads to the overproduction of O_2_•^−^ and exacerbates oxidative stress [59]. Regenerated ROS further damages the mitochondria, leading to enhanced free radical production and severe loss of function of the antioxidant system, eventually creating a vicious cycle that exacerbates oligodendrocyte apoptosis and demyelination.

Neuronal and axonal degeneration leading to disability, which is an important neurodegenerative marker of MS, is closely associated with oxidative damage [60]. Neurons predominantly express N-methyl-D-aspartic acid (NMDA) receptors in the CNS. In addition, glutamate in MS continuously activates the NMDA receptors [61], leading to a massive Ca2+ influx through the NMDA receptor-mediated channels. The increase in intracellular calcium levels leads to the production of NO and O_2_•^−^ by neuronal nitric oxide synthase (nNOS) and mitochondrial stimulation, respectively [62]. The massive production of NO and O_2_•^−^ leads to the production of ONOO^−^. Furthermore, ONOO^−^ is the most common toxic effector molecule in MS [63], resulting in protein nitration and lipid peroxidation [64]. Moreover, ONOO^−^ promotes the nitration of tyrosine hydroxylase [65], mitochondrial SOD [66] and heat shock protein 90 (HSP90) [67], interferes with mitochondrial function and induces apoptosis in neuronal cells [68].

When neurons are exposed to an inflammatory microenvironment, pro-inflammatory cell-derived ROS can induce oxidative damage, reduce mitochondrial enzyme activity, and lead to mitochondrial DNA damage, thereby decreasing neuronal energy metabolism. Recent studies have revealed that oxidative phosphorylation (OXPHOS) is involved in neuronal damage in MS [69], which is co-regulated by multiple transcription factors [70]. The expression of transcription factor complexes containing nuclear respiratory factor 2 (Nrf2) in nuclear extracts isolated from the cortex of patients with MS was found to be attenuated compared to normal subjects [71]. This attenuation was associated with lowered OXPHOS gene expression level and increased oxidative stress.

Overexpression of ROS can regulate Nrf2 through a transcription-mediated mechanism, thereby reducing Nrf2 binding to decrease neuronal energy metabolism. Peroxisome proliferator-activated receptor γ coactivator-1α (PGC-1α), a transcriptional cofactor, activates nuclear transcription factors involved in mitochondrial function [72]. The expression level of PGC-1α is significantly reduced in the cingulate gyrus and frontal cortex in patients with MS, leading to extensive neuronal loss (positive correlation) [73]. Reduced PGC-1α expression in neuronal cells results in attenuated transcription of OXPHOS [74] subunits and mitochondrial antioxidants [75]. Furthermore, PGC-1α knockdown results in decreased mitochondrial membrane potential, enhanced ROS formation and increased susceptibility to free radical-induced cell death in neurons [76]. Therefore, energy depletion caused by oxidative and mitochondrial damage in MS is crucial for neuronal dysfunction and death.

## 3. Oxidative Stress in Animal Models of MS

Rodents are widely used in pre-clinical animal models of MS due to their similar physiology to humans and the advantages of low experimental costs and well-established study protocols. In particular, mice and rats are commonly used for in vivo studies of MS-related pathological changes, molecular mechanisms and pharmacological evaluation.

Experimental autoimmune encephalitis (EAE) is the most effective and commonly used model to explore the various characteristics, pathogenesis and novel therapeutic agents of MS. EAE is an autoimmune disease characterized by monocyte infiltration and demyelination at the periphery of small blood vessels in the CNS [77,78]. It is a classical experimental animal model of MS [79]. The most common model of active sensitization in mice is induced by complete Freund’s adjuvant (CFA), combined with subcutaneous injection of myelin peptide fragments. CFA is composed of heat-killed Mycobacterium tuberculosis H37Ra strain suspended in paraffin oil. It can prolong the retention period of antigen in the body, promotes the formation of high-affinity antibodies, and strengthen the inflammatory reaction at the antigen injection site, which is conducive to stimulating the proliferation of immune cells [80]. Besides, CFA contains pathogen-associated molecular patterns (PAMPs) that bind toll-like receptors (TLRs) and is necessary to induce EAE [81]. The myelin peptide fragment is recognised by T cells, resulting in increased activity of myelin protein-reactive CD4^+^ Th1 cells [82]. In addition, intraperitoneal injection of pertussis toxin [83], which has a blood–brain barrier (BBB) disrupting effect, allows the peripheral reactive T cells to enter the CNS [84], leading to an inflammatory response and demyelination [85]. Myelin antigens induce different strains of animals to produce specific autoreactive T cells against the antigens to mimic the various subtypes of MS in the EAE model [86]. For example, MOG_35–55_-immunized C57 mice and PLP_139–151_-immunized SJL mice can induce chronic-relapsing and relapsing-remitting EAE, respectively [87]; MOG_92–106_-induced ASW mice can simulate primary-progressive MS [88]; and MBP_68–82_-induced Lewis rats are commonly used to simulate acute attacks of MS [89]. In addition, encephalitis-causing specific T cells isolated from the peripheral immune organs of animals sensitised by myelin antigens can be transferred to another group of recipient animals (passive immunization), during which the T cells cross the blood-cerebrospinal fluid barrier to trigger inflammation in the brain [90,91]. Passive EAE can be considered to study the direct induction of the pathogenesis by CD4^+^ T cells in the absence of adjuvants [92], demonstrating that these cells play a key role in the pathogenesis of EAE. In this model, inflammation is mediated by T cells, accompanied by microglia and macrophage recruitment and activation.

Regulatory T cells (Tregs) are essential for maintaining immune homeostasis, and their dysfunction is a major feature of autoimmune diseases [93]. Overproduction of mitochondrial ROS (mtROS) within Tregs and the resultant sustained inhibition of lysosomal function in mice with EAE impair the mitochondrial autophagic pathway to enhance mitochondrial oxidative stress and DNA damage response, eventually promoting cell death [94]. A study explored oxidative stress in the CNS of rats with early-stage EAE and observed a decrease in the mitochondrial membrane potential in the brain and spinal cord on days 5–7 after immunization. This depolarization across the inner membrane may indicate an impaired electron transport chain, leading to proton loss. The collapse of the mitochondrial membrane potential is closely related to intracellular redox homeostasis. The oxidation levels in the brain, spinal cord and optic nerve have revealed a significant increase in the number of carbonyl proteins and nitration of tyrosine residues in the brain tissues of rats with EAE starting from day 7 after immunization [95]. In rats with chronic-relapsing EAE, two peaks were observed on days 17 and 32 after immunization. An extensive activation of microglia in the rat brain was observed in both the first and the second attacks. During this period, elevated levels of MDA, glutathione reductase and cholesterol, as well as decreased CAT, GSH and desmosterol activity were observed in the hippocampus [96], ultimately leading to massive apoptosis of the neuronal cells. During clinical recovery from chronic MS, demyelination and neurodegeneration progress even in the absence of significant immune cell infiltration. Chronic persistent oxidative stress is the primary cause of subclinical neuronal dysfunction in the recovery phase of EAE. At the peak of EAE, large amounts of activated NADPH oxidase have been detected in the mouse brainstem, leading to increased oxidative stress. During the recovery phase of EAE, local activation of NADPH oxidase persists even when the inflammatory response becomes mild. Furthermore, during the remission phase of EAE, ROS-mediated oxidative damage is mainly derived from chronically activated microglia and astrocytes, both of which participate in oxidative stress generation by more than 70% [97].

Cuprizone (CPZ), a copper ion chelator, can induce demyelination through oxidative damage. The pathology of this model is characterized by primary demyelination and the absence of a T cell-mediated inflammatory response, which induces extensive activation of microglia and astrocytes [98]. Chelation of copper ions by CPZ leads to a dysfunction of the mitochondrial complex IV [99] in oligodendrocytes and inhibition of a series of enzymes affecting copper ions, such as mitochondrial enzymes, cytochrome oxidase and monoamine oxidase, thereby inducing apoptosis of oligodendrocytes [100]. CPZ induces both acute and chronic demyelination. In addition, C57BL/6 is by far the strain with the highest incidence of demyelination. However, the use of this strain is influenced by sex and age, and 6–9-week-old female mice are commonly used. An acute demyelination model of MS can be established by feeding mice a diet containing 0.2–0.3% CPZ for 4–6 weeks. This model is characterized by spontaneous remyelination in the white matter regions such as the corpus callosum after the cessation of CPZ. Therefore, this model is commonly used in drug development to promote myelin repair [101]. In addition, a chronic demyelination model can be developed after exposing the animals to CPZ for 12 weeks. Minimal remyelination occurs after chronic demyelination [102,103]. Therefore, this model is mainly used to study long-term demyelination associated with MS. One study reported that CPZ-induced demyelination alters the mitochondrial electron transport chain. After 3 weeks of CPZ treatment, the mitochondria within oligodendrocytes swelled and elongated, and giant mitochondria appeared, leading to insufficient energy production. Subsequently, this led to the destruction of mitochondria, resulting in the generation of large amounts of free radicals [104] within the CNS. During this period, the level of GSH, an antioxidant, was significantly reduced, whereas the expression level of the oxidised form of GSH, GSSG, increased several-fold. In addition, glycerophosphate dehydrogenase (GPDH) plays a role in the maintenance of cellular redox potential. After CPZ treatment, GPDH expression was found to increase significantly due to the cellular response to increased oxidative stress [105]. Furthermore, in vitro exposure of oligodendrocytes to CPZ reduces intracellular CAT activity and increases the levels of MDA and lipid peroxidation [106].

Lysophosphatidylcholine (LPC), the major component of oxidized low-density lipoprotein [107], has a specific affinity for oligodendrocytes without significant disruptive effects on neuronal axons [108]. Therefore, it is used to simulate focal demyelination in the CNS. This model is usually created by direct local injection of 1% LPC, which is specifically toxic to the myelin proteins and induces fusion of the myelin lamellae, transformation into spherical vesicles and its progressive shrinkage until phagocytosis [109]. Injecting LPC into the white matter region of the CNS leads to massive microglial activation, reactive astrocyte proliferation and oligodendrocyte damage [110]. Myelin undergoes spontaneous repair and regeneration after 7 days of LPC injection [111]. This model is unique in its spatial and temporal control of demyelination and is a highly reproducible model of focal primary demyelination. Therefore, it is often used to study the complex mechanisms of remyelination. Furthermore, LPC is an activator of phospholipase A2 [112], a central mediator of the inflammatory response in MS [113]. LPC can recruit macrophages, leading to excessive production of pro-inflammatory cytokines, NO and free radicals, thus exacerbating oxidative stress in demyelinating lesions. Injection of LPC into the rat hippocampus was found to significantly increase the level of the lipid peroxidation marker MDA and significantly decrease the activity of the peroxidation marker CAT [114]. Injection of LPC into the optic nerve of rats strongly induced the expression of iNOS and affected the Nrf2 and Heme oxygenase (HO)-1 pathway. In vitro experiments confirmed that LPC treatment of OLN-93 oligodendrocytes causes calcium influx, resulting in a decrease in the mitochondrial membrane potential, the production of large amounts of mtROS and RNS and high expression of nNOS, NO and lactate dehydrogenase (LDH) [115].

Demyelination caused by the local injection of ethidium bromide (EB) into the white matter of the CNS is also one of the commonly used models of toxic demyelination. EB, initially used as a staining agent in DNA gel electrophoresis, embeds in chromosomes and mtDNA, prevents the replication and transcription of mtDNA and inhibits mitochondria-associated RNA, leading to cell death. The optimal injection dose and concentration of EB are 0.2 μL and 5 μg/mL, respectively. A 3-day injection of EB into the brain triggers apoptosis of oligodendrocytes, producing focal demyelination and strong [116] activation of the microglia and astrocytes [117]. Similar to the LPC model, the EB model has significant advantages to study the site-specific demyelination and remyelination processes, and the extent of damage can be controlled by adjusting the concentration. In addition, intracerebral injection of EB significantly increases oxidative damage in rat brain tissue, as evidenced by upregulation of MDA and NO and downregulation of GSH, GPx and AchE. Furthermore, EB intervention in the SH–SY5Y cell line was found to result in the disruption of intracellular mitochondrial DNA strands and inhibition of SOD, CAT and γ-glutamylcysteine synthetase (GCS) activities [118].

Specific viruses have been used to induce MS due to their association with the onset and progression of infections. Studies have revealed that Theiler’s murine encephalomyelitis virus (TMEV), canine distemper virus (CDV) and mouse hepatitis virus (MHV) can be used to construct animal models of virus-induced demyelination. TMEV, a neurotropic single-stranded RNA virus that persistently infects the murine central nervous system [119], primarily affects the spinal cord. TMEV infection causes axonal damage before demyelination [120]. Specifically, axonal damage can lead to the destruction and release of myelin proteins, inducing an autoimmune response against myelin antigens and allowing T cells and macrophages to enter the CNS, thereby causing myelin damage. The presentations of TMEV-mediated demyelination are similar to the clinical signs of MS. Furthermore, the autoimmune response to TMEV infection is largely consistent with that of MS: the inflammatory infiltrate within the demyelinated plaques consists of CD4^+^ cells, CD8^+^ T cells [121], B cells [122] and plasma cells [123], and a large number of activated microglia are present in the region of the lesions. Therefore, the TMEV model has similar characteristics as those seen in clinical settings (in relation to the EAE-induced MS model) for simulating chronic progressive MS. After 1 month of TMEV infection, the affected mice were found to exhibit weakness, spasticity, and in severe cases, tonic spasms, in addition to slowly progressive inflammatory demyelination [124]. Currently, the TMEV model is being widely used to study the mechanism of virus clearance in the CNS. Moreover, this model can be used to determine the role of different lymphocyte populations in the course of MS. A study reported that 72-h treatment of TMEV in the mouse brain resulted in the reduction of GSH levels, increased the expression of GSSG, and significantly downregulated the GSH/GSSG ratio in the hippocampus. As expected, intense oxidative stress was observed in the hippocampus of TMEV-infected mice. A marker of protein nitration, 3-nitrotyrosine (3-NT), is a post-translational modification that leads to protein dysfunction. A peroxynitrite-specific attack on the tyrosine residues of proteins leads to the formation of 3-NT. A substantial increase in 3-NT levels was observed in TMEV-infected mice on days 3, 4 and 14. Thus, protein nitration is upregulated in the brains of TMEV-infected mice, suggesting increased production of ROS and RNS [125].

MHV is another virus that induces demyelination in the CNS. After MHV infection, viral RNA and virus-specific T cells persist in the nervous system of mice for life. Infected mice present clinical signs such as hind-limb weakness, clumsy gait and paralysis [126]. Demyelinating lesions peak 1 month after injection of the virus. Early studies reported that MHV could directly infect oligodendrocytes, thereby triggering cell damage and demyelination [127]. However, immune-mediated oligodendrocyte damage is the main mechanism of this virus-induced demyelination along with the pathological manifestations of a chronic inflammatory response, primary demyelination and axonal damage [128]. MHV-induced demyelination is characterized by infiltration of T lymphocytes and macrophages [129]. In the MHV model, massive activation of monocyte-derived macrophages and microglia were observed in the brain and spinal cord in contact with the damaged myelinating axons, along with the release of oxygen radicals [130]. Accumulation of ROS in the optic nerve increased significantly 30 days after intracranial inoculation of MHV in mice. In addition, the levels of succinate dehydrogenase (SDH), SOD and PGC-1α in the optic nerve and retinal mitochondria of mice were suppressed, and demyelination and neuronal loss in the optic nerve and spinal cord were effectively induced [131].

## 4. Natural Compounds against Oxidative Stress in Animal Models of MS

The antioxidant effects of natural products in MS have been extensively studied in recent years. Natural products have been used in rodent models of MS and have been found to reduce CNS and peripheral immune-inflammatory responses, alleviate demyelination and axonal degeneration, and attenuate oxidative stress damage in the brain, spinal cord and optic nerve, thereby ameliorating various clinical symptoms of MS. The mechanisms of antioxidant and neuroprotective effects of these natural products in animal models of MS (rats and mice) are summarized in Table 1.

### 4.1. Natural Phenolic Compounds

Natural phenolic compounds are plant-derived antioxidants. Phenols, the important secondary metabolites in plants, are mainly produced by the metabolic pathways of shikimic acid [132]. Phenols are widely found in the roots, stems, leaves, flowers, fruit pulp and seeds of various higher plants. Among the major bioactive substances of plants, phenolic compounds are the most diverse and widely studied class of chemicals. Structurally, all phenolic compounds contain at least one benzene ring and one hydroxyl group. The strong antioxidant capacity of natural phenols is mainly attributed to the hydroxyl group, which can effectively quench free radicals, chelate metal ions, induce antioxidant enzyme activity and regulate the in vivo antioxidant signalling pathways [133]. Natural phenolic compounds can be classified into flavonoids and non-flavonoids depending on their chemical structures.

#### 4.1.1. Flavonoids

Anthocyanin (ANT), a flavonoid, is one of the polyphenolic compounds with the highest concentration in food. Natural ANT, widely found in angiosperms, are water-soluble natural pigments that give petals and fruits purple, red and blue colors. ANT have anti-inflammatory, anti-tumour, anti-ageing, and metabolism-regulating effects, which are related to their excellent antioxidant capacity [134]. The antioxidant mechanisms of ANT mainly include the reduction of ROS accumulation and scavenging of free radicals, activation of the enzymatic antioxidant system and attenuation of DNA damage. Oral administration of ANT to rats with EB-induced MS was found to reduce demyelination, decrease the levels of HNE-histidine (HNE-His) and MDA and increase the activities of Non-protein sulfhydryl group (NPSH), GSH and SOD. Administration of ANT also resulted in near-normal levels of protein carbonylation, NOX and inflammatory cytokines in rats with EB-induced demyelination. In addition, EB increased MPO activity, and MPO expression was associated with inflammatory cell infiltration. The presence of neutrophils and the release of active substances can lead to the increase of oxidative stress, the decrease of ion pump activity, and the death of oligodendrocytes. ANT treatment partially inhibited the aggravation of interleukin-induced inflammation and decreased EB induced ion pump activity. Treated with ANT also reduced MPO and inflammatory cell infiltration in rat brain tissue, thus preventing the harmful effect of EB on demyelination [135].

Hesperidin (HP), a natural flavonoid widely found in the plant kingdom (mainly in citrus plants), is the main pharmacological component of the fruits of the genus *Citrus*, family Rutaceae, with various biological activities including anti-inflammatory, antioxidant, and tumour metabolism regulatory functions [136]. EAE-induced lipid peroxidation caused a significant increase in TBARS levels, leading to irreversible CNS injury. In addition, EAE inhibits the enzyme (SOD, CAT, and GPx) and non-enzyme (GSH) antioxidant defense systems in brain tissue. Oxidative stress plays an essential role in MS because it can lead to significant cell death and neuronal damage in the brain. In this case, the effect of EAE on the brain may mainly depend on the increase of oxidative stress. Therefore, antioxidants such as HP may be helpful for EAE-induced demyelination. Intraperitoneal injection of HP after the onset of EAE reduced the disease severity by decreasing the levels of interleukin (IL)-17, IL-1β and tumor necrosis factor (TNF)-α, inhibiting lipid peroxidation (TBARS) and increasing the antioxidant markers (GSH, CAT, GPx and SOD) in brain tissues, thereby reducing histopathological damage in the brain [137].

Licochalcone A is a flavonoid extracted from liquorice roots and is a species-specific component of *Glycyrrhiza inflata*. Licochalcone A is the most abundant among the chalcones isolated and identified in liquorice. Compared with the normal control group, spleen cells from the EAE group showed high levels of oxygen free radicals (H_2_O_2_, NO) and cytokines (TNF-α, IFN- γ, and IL-17). As observed in MS and EAE, long-term exposure to oxidative stress environment will destroy the biological structure and lead to significant cell destruction, causing direct damage to the myelin sheath. It has been reported that IFN- γ (Th1 cytokine), IL-17 (Th17 cytokine), and TNF- α play a vital role in the induction and severity of MS/EAE. In the acute phase of EAE, the expression level of IFN- γ and TNF- α was parallel to the severity of clinical symptoms and the degree of inflammatory cell infiltration. Licochalcone A can alleviate the rise in clinical scores and weight loss involved in EAE and terminates the excessive immune response, which is attributed to its property of inhibiting the levels of interferon (IFN)-γ, TNF-α and IL-17. In addition, it can reduce the expression of the oxidative markers H_2_O_2_ and NO, as confirmed by both in vitro and in vivo experiments [138].

Flavonoids in *Scutellaria baicalensis* and *Acacia catechu* exert a variety of therapeutic effects, including anti-inflammatory, antiviral, antibacterial, and anticancer activities. Unlike traditional natural flavonoids, flavocoxid (FVC) is a purified mixture containing baicalin and catechin that acts as a dual inhibitor of cyclooxygenase (COX)-2 and 5-lipoxygenase (LOX) [139]. Th1/Th17 cells play an essential role in EAE. These cells migrate from the spleen to the CNS. They are re-stimulated by perivascular antigen-presenting cells (APCs) and promote microglia activation by releasing pro-inflammatory cytokines. Activated microglia have two different phenotypes, the classical M1 and the alternatively activated M2. M1 cells express iNOS, produce high levels of oxidative metabolites and pro-inflammatory cytokines, and destroy myelin sheath. M2 phenotype has neuroprotective and anti-oxidative effects. In addition to its anti-inflammatory properties, FVC serves as an antioxidant to reduce ROS, including •OH, O_2_•^−^ and H_2_O_2_. FVC was shown to attenuate the severity of EAE, reduce the levels of COX-2, 5-LOX, iNOS, IL-12, IL-23, IL-17 and IFN-γ and increase the levels of IL-10 in the spinal cord of mice. In addition, oral administration of FVC decreased the M1 phenotypes and increased the proportion of M2 phenotypes in macrophages and microglia. Furthermore, FVC inhibited CD4^+^ T cell Th1/Th17 differentiation [140].

Luteolin was originally isolated from the stems and leaves of the mignonette plant. Recent studies have revealed that luteolin is predominantly present as glycosides in a variety of herbs, vegetables and fruits, such as *honeysuckle*, *Schizonepeta* and pomegranate. Luteolin has multiple pharmacological properties such as antibacterial and antiviral properties. In addition, it has exhibited excellent safety and efficacy in a variety of pre-clinical models as an anti-inflammatory, antioxidant and neuroprotective agent [141]. Furthermore, luteolin is a viable therapeutic agent for oxidative stress associated with autoimmune diseases. NF-κB activation in MS patients leads to extreme expression of chemokines and cytokines, macrophage inflammatory protein-1α (MIP-1α), one of the most frequently expressed chemokines throughout CNS inflammation, involved in MS/EAE progress. Neuronal and oligodendrocyte apoptosis is considered an important feature of MS, which may be attributed to oxidative stress-induced cell membrane damage and impaired myelination, and activation of caspase-3 within neurons leads to axonal degenerative changes. cAMP has shown a critical role in neuronal growth, differentiation and survival. cAMP levels were decreased in the cerebrospinal fluids of MS patients, and inhibition of the cAMP/CREB signaling axis attenuated CNTF expression. Compared to EAE mice, luteolin-treated EAE mice demonstrated improved clinical motor function, reduced inflammatory infiltration of brain tissues, upregulated ciliary neurotrophic factor (CNTF) expression, significantly increased levels of cAMP and total antioxidant capacity (TAC) of the brain and significantly reduced levels of cleaved caspase 3, nuclear factor kappa-B (NF-κB) and macrophage inflammatory protein (MIP)-1α [142].

Epimedium flavonoids (EF) are a total flavonoid component extracted from the stem and leaves of plants belonging to *Epimedium* spp. of the family Berberidaceae, containing mainly icariin and icariside. EF exhibited good neuroprotective effects in animal models of Alzheimer’s disease, traumatic brain injury, ischaemic stroke and depression. Microglia and astrocytes were activated in the brain and spinal cord of EAE model, resulting in the production and release of pro-inflammatory molecules (IL-1β, TNF-α, NO) and the upregulation of iNOS activity. NF-κB is a family of transcription factors critical for immunity and inflammation. It regulates many biological processes, including sensitivity to inflammatory cytokines, oxidative stress, and apoptosis. NF-κB-dependent CNS immune cell activation and oligodendrocyte death are essential pathophysiological features of multiple sclerosis. Oral administration of EF to rats with EAE resulted in the attenuation of neurological deficits, demyelination and inflammatory infiltration, activation of astrocytes in the spinal cord, and inhibition of IL-1β, TNF-α and NF-κB expression. In addition, it resulted in upregulated level of nerve growth factor (NGF) and improved the ultrastructure of myelin and axons. Furthermore, oxidative stress-related iNOS and NO in the spinal cord of rats with EAE were downregulated after treatment with EF [143].

Kolaviron (KV), derived from traditional African medicine, is a bioflavonoid isolated from the seeds of Garcinia kola. Previous in vitro and in vivo studies have revealed that KV has many pharmacological properties such as anti-inflammatory, antibacterial, anti-apoptotic and antioxidant properties [144]. CPZ significantly downregulated SOD levels within the rat prefrontal cortex, exacerbated lipid peroxidation, reduced spatial memory and disrupted the integrity of neuronal and non-neuronal cells. Moreover, during CPZ intervention, GSH content decreased, and excessive ROS/RNS were produced to induce CNS damage. CPZ poisoning can lead to the formation of giant mitochondria and oxidative stress, resulting in the interruption and shortage of energy flow. The abnormal energy flow impairs the function of the endoplasmic reticulum, causes extensive proliferation of the endoplasmic reticulum and decreases hydrophobin synthesis, aggravating the disintegration of oligodendrocytes and myelin sheath. However, these changes were significantly reversed in KV-treated rats. Such anti-demyelinating pharmacological effects of KV are mediated by its inherent antioxidant properties to counteract CPZ-induced oxidative damage. Therefore, KV, a natural compound with neuroprotective effects, deserves further study as a potential anti-MS therapeutic agent [145].

Quercetin (Que), a polyhydroxy flavonoid, is a secondary plant metabolite that is widely found in a variety of vegetables and fruits such as hawthorn, broccoli and onions, as well as in herbs such as *Forsythiae Fructus*, *Gynostemma pentaphyllum* and *Houttuynia cordata*. Que exhibits prominent antioxidant, free radical scavenging, anti-inflammatory and immunomodulatory properties [146]. The researchers found that the demyelination of pons caused the decrease of Na^+^, K^+^-ATPase activity, directly damaging axonal pulses’ transmission. The measurement of blood biomarkers may be one of the crucial methods to evaluate the oxidative status and disease severity of neuroinflammation. In some CNS inflammatory diseases, oxidative stress and inflammation interact with redox dysfunction in erythrocytes. Erythrocytes in MS patients are more prone to cytolysis, and erythrocyte membrane fluidity defects are closely related to the intensity of demyelination and the process of lipid peroxidation. In rats with EB-induced demyelination, treatment with Que increased Na^+^ and K^+^ ATPase activity in the pons and cerebellum and inhibited the EB-induced increase in acetylcholinesterase activity in whole blood and lymphocytes. EB decreased the activity of CAT in whole blood and increased serum MDA levels in rats. However, Que reversed this change, significantly increased the antioxidant capacity and alleviated the EB-induced inflammatory cell infiltration and behavioural deficits in rats [147].

Phloretin (Phl) is a dihydrochalcone compound found in common fruits such as apples and strawberries. Phl has immunomodulatory properties and has been widely studied for its natural antioxidant properties. In vivo studies have revealed that Phl alleviated the clinical signs and neuroinflammation in mice with EAE. Nrf2 is a significant regulator of antioxidant response. Many studies have determined that its activation can drive macrophages to produce anti-inflammatory phenotypes and reduce neuroinflammation and neurodegeneration in CNS diseases. Nrf2 activation is mediated by AMPK activation, AMPK plays a crucial role in restoring cellular energy homeostasis. It has been found that Phl stimulates autophagy in an AMPK-dependent manner and activates the Nrf2 pathway through autophagy-mediated degradation of Keap1 to reduce macrophage-derived NO and ROS, ultimately driving macrophage polarisation toward a protective phenotype. These findings suggest that Phl may be a potential therapeutic agent for inflammatory demyelinating diseases of the CNS [148].

#### 4.1.2. Non-Flavonoids

Curcumin extracted from turmeric root is a natural polyphenolic compound with multiple bioactivities that has been applied in a variety of neurodegenerative diseases. Curcumin was found to effectively improve neurobehavioural deficits, weight loss and demyelination of the corpus callosum in mice with EAE. These effects were attributed to the inhibition of mRNA levels of IL-6, IL-17, TNF-α and IFN-γ in brain tissues and increased activity of the antioxidant enzymes SOD and GPx [149]. Polymerised nano-curcumin enhanced neural and myelin repair in rats with EAE by increasing the levels of the anti-inflammatory factors IL-4, IL-10 and transforming growth factor (TGF)-β in the CNS and upregulating the HO-1/Nrf2 antioxidant pathway. In addition, iNOS was significantly inhibited in the spinal cord tissue of curcumin-treated rats with EAE [150] Therefore, curcumin may play a neuroprotective role in EAE mice by down-regulating pro-inflammatory cytokines, up-regulating the expression of anti-inflammatory cytokines, and enhancing the antioxidant defense system in the brain and spinal cord.

Resveratrol (RV), a natural non-flavonoid polyphenolic compound extracted from plants, is an antitoxin secreted by the plant in response to a pathogenic attack. RV is widely found in many common plants, such as grapes, peanuts, mulberries and *Veratrum nigrum* L. RV has a variety of pharmacological effects such as anti-inflammatory, anti-infective and immunomodulatory effects and is widely used in inflammatory demyelinating diseases of the CNS. The increase of blood–brain barrier permeability is one of the main characteristics of EAE. Due to the destruction of basement membrane tight junction protein, various proinflammatory factors and oxygen free radicals enter the brain through the damaged BBB, resulting in brain edema, demyelination and nerve cell death. RV has been reported to inhibit EAE-induced overexpression of iNOS and IL-1β and upregulate Arg-1 and IL-10 expression in the brain [151]. In addition, RV has been found to reduce the overexpression of NOX2 and NOX4 and inhibit NADPH oxidase activity. Furthermore, RV ameliorated the clinical severity of MS by maintaining the integrity of the blood–brain barrier in mice with EAE by reducing oxidative damage. It is reported that the demyelinating brain tissue induced by cuprizone involves mitochondrial damage, showing the destruction of electron transport chain and the obstruction of oxidative phosphorylation. In a CPZ-induced demyelination model in mice, RV significantly alleviated oxidative stress, corrected the abnormal expression of peroxidation products (LPO), GSH and SOD, improved mitochondrial function, reversed demyelination and promoted remyelination [152].

Acteoside (AC) is a water-soluble phenylpropanoid glycoside that commonly exists in plants like *Rehmanniae Radix*, *Cistanches Herba*, and *Plantaginis Semen*. Modern pharmacological studies show that AC has significant antioxidant, anti-inflammatory, neuroprotective, antitumor, anti-pathogenic microbial and other effects. Due to its comprehensive source, high biological activity and low adverse effects, AC’s medicinal value is gaining more and more attention. AC could significantly inhibit inflammatory response and demyelination in the CNS of EAE mice. Mitochondrial dysfunction is an important factor in the development of MS/EAE. Free radicals mainly originate from damaged mitochondria, and mitochondrial proteins are vulnerable to ONOO- induced nitrification stress. ONOO-mediated excessive mitophagy recruits Drp1 to damaged mitochondria, exacerbating axonal degeneration and neuronal cell death. Therefore, targeting ONOO-induced mitophagy may be a new therapeutic approach for MS. AC also reduced the production of ONOO^−^, down-regulated iNOS and NADPH oxidase, and inhibited apoptotic cell death and mitochondrial damage in the spinal cord of EAE mice. In vitro studies further demonstrated that AC could scavenge ONOO^−^ and protects neuronal cells from nitrative cytotoxicity by inhibiting ONOO^−^ mediated excessive mitochondrial autophagy [153].

Oleacein (OLE) is a cyclic enol ether terpene polyphenol found in *Olea europea* L. and *Ligustrum vulgare* L., which is a common nutrient in the Mediterranean diet. Many studies have defined OLE as a beneficial compound for health and it has potent antioxidant activity in the treatment of neuroinflammatory diseases. OLE treatment was effective in reducing clinical scores and histological damage in EAE. Leukocyte infiltration, demyelination, blood–brain barrier destruction and superoxide anion accumulation were reduced in CNS tissues of EAE mice treated with OLE. Previous evidence suggests that (granulocyte-macrophage colony-stimulating factor) GM-CSF, which is predominantly expressed by helper T cells, plays a critical role in assisting peripheral immune cell migration across the blood–brain barrier. Once GM-CSF enters the CNS, it supports immune cell differentiation into a pro-inflammatory phenotype and produces Numerous neurotoxic mediators, including IL-1β, IL-6, TNFα and ROS, which are involved in tissue damage during EAE. Elevated expression of GM-CSF is a fundamental cause of inflammatory cell infiltration and demyelination in the CNS of EAE mice. OLE significantly reduced the expression of GM-CSF, IL-13, TNF-α, MCP-1 and IL-1β, while increasing IL-10. At the same time, OLE significantly upregulated the ROS interferer Sestrin-3. Mechanistically speaking, OLE blocked the expression of NLRP3, phosphorylation of p65-NF-*κ*B, and reduced the synthesis of pro-inflammatory factors in BV2 cells. In addition, OLE also prevented RGC-5 cell apoptosis induced by oxidative stressors [154].

Ellagic acid (EA) is a powerful natural antioxidant. It is a dimeric derivative of gallic acid found in many fruits, vegetables, and herbs and is widely used in food, cosmetics, and pharmaceuticals [155]. EA rarely exists free in nature but is usually present in plants as ellagitannins, which can be hydrolyzed to ellagic acid by intestinal flora after ingestion by animals. Increased ROS production in serum and cerebrospinal fluid, oxidative phosphorylation dysfunction, calcium homeostasis imbalance, and decreased antioxidant defense systems have been reported in MS disease. Cuprizone reduces antioxidant activity by chelating copper, resulting in significant oxidative damage, neurotoxicity and demyelination. CPZ-induced demyelination increased ROS levels and MDA content in spinal cord tissue, and decreased SOD and CAT activity. The oral administration of EA significantly reversed these abnormal changes. EA treatment exerted spinal cord neuroprotective effects by alleviating oxidative damage, reducing demyelination severity, and improving behavioral impairment in CPZ mice [156].

Arbutin is a natural soluble glycosylated phenol isolated from the leaves of *Vaccinium vitis-idaea* L., Rhododendron family member, with antioxidant, anti-microbial infection, anti-inflammatory and anti-apoptotic effects. Animal experiments in vivo have found that arbutin can improve many diseases of urinary system, circulatory system, endocrine system and nervous system [157]. Arbutin has long-lasting free radical scavenging properties and reduces oxidative stress by reducing the production of reactive oxygen species and superoxide. Arbutin can significantly reduce delayed LPC-induced visual evoked potentials and demyelination, down-regulate IL-1β, IL-17, TNF-*α* and iNOS mRNA levels in LPC rat brain tissue, and enhance the expression of IL-10, Nrf2 and HO-1. In addition, the researchers found that the injection of LPC induced the activation of neuroglia in the demyelinating area of CNS, and reactive astrocytes caused the production of a large number of free radicals and intense oxidative stress in the lesion area, which aggravated the death of oligodendrocytes in the optic nerve. Arbutin attenuated the astrocyte marker GFAP in the brain. These results suggested that arbutin improves myelin repair and functional recovery of demyelination of the optic nerve by attenuating inflammation and oxidative stress [158].

Paeonol is a natural phenolic compound derived mainly from the roots of *Paeonia suffruticosa* and *Cynanchum paniculatum*. The pharmacological effects of paeonol are numerous. The neuroprotective effects of paeonol as an antioxidant shown in neurodegenerative diseases such as Parkinson’s and Alzheimer’s have received increasing attention in recent years [159]. Copper is an important cofactor for superoxide dismutase production. CPZ-induced copper deficiency in the CNS has deleterious effects on the endogenous antioxidant system, leading to oxidative stress and lipid peroxidation in the brain. CPZ increased hippocampal levels of MDA and ROS, and decreased GSH, SOD, and CAT activity. CPZ also leads to the massive release of neuroinflammatory mediators in the mouse CNS by disturbing the expression of NF-κB and IκB-α. One study found that paeonol treatment attenuated deficits in Y labyrinth, Barnes labyrinth, and new object discrimination tests in CPZ mice, increased MBP expression in demyelinated regions, and reversed abnormal expression of MDA, ROS, GSH, SOD, CAT, NF-κB, and TNF-α. In addition, paeonol improved CPZ-induced depolarization of mitochondrial membrane potential. In conclusion, in addition to maintaining the mitochondrial function, paeonol reduced oxidative stress and inflammation-related indicators to alleviate CPZ-induced demyelination and cognitive deficits [160].

### 4.2. Terpenoids

The plant constituents formed by the derivation of mevalonate are called terpenoids. Terpenoids are the most numerous and diverse natural products, which can be classified into monoterpenes, sesquiterpenes, diterpenes, triterpenes, and polyterpenes according to their chemical structures. These compounds have numerous biological activities, including anti-inflammatory, antioxidant and immunomodulatory, and are widely used in various diseases, which have attracted increasing attention in recent years [161].

*Glycyrrhizae Radix et Rhizoma* is a commonly used herb in traditional Chinese medicine. 18β-glycyrrhetinic acid (GA) is a pentacyclic triterpene derivative isolated from *Glycyrrhizae Radix et Rhizoma,* which is a hydrolyzed metabolite of glycyrrhizinic acid and liquiritigenin. Due to its antioxidant, anti-inflammatory and anti-cancer effects, GA is often used to treat digestive and neurological disorders. As we all know, inflammation is significant in autoimmune diseases such as MS. The excessive production of pro-inflammatory cytokines and chemokines will cause the death of nerve cells and lipid membrane disorder in the CNS of EAE mice, which lead to the generation of reactive oxygen species, aggravating the oxidative damage of neurons and glial cells. EAE induced a significant increase in oxidative stress and lipid peroxidation (elevated TBAR levels, decreased GPx, SOD, CAT and GSH levels) in the mouse brain. In addition, EAE mice had many inflammatory cells infiltrating and highly expressed pro-inflammatory factors and apoptosis markers (IL-17, TNF-*α* and IL-1*β*, Caspase-3). In contrast, GA treatment significantly reversed the oxidative histological and pathological changes of EAE [162]. *β*-Caryophyllene (BCP), a natural sesquiterpene found in many plants, is a potent anti-inflammatory compound. In MS, when microglia and macrophages are activated, they release many cytotoxic mediators, including IFN-γ, TNF-α, NO, and ROS, which may cause CNS tissue damage. IFN-γ is involved in macrophage activation, T cell differentiation, and regulation of T lymphocyte function, which is particularly critical in autoimmune diseases. TNF-α is an important factor causing neuronal damage, and its production is regulated by NF-κB. Animal studies revealed that BCP treatment reduced the clinical severity and pathological damage of EAE and inhibited the production of TNF-*α*, IFN-*γ* and IL-17 as well as oxidative markers H_2_O_2_, and NO in CNS. Consistent with the in vivo results, in vitro intervention with BCP in splenocytes of EAE mice significantly reduced oxygen free radicals and inflammation [163].

Ginkgolide K (GK) is a diterpene lactone compound extracted from the leaves and roots of Ginkgo, which has antagonistic properties against platelet-activating factor receptors. Evidence suggests that Ginkgo extracts have neuroprotective effects under hypoxia/ischemia, Alzheimer’s disease, and anxiety [164]. Intraperitoneal injection of GK improved behavioral dysfunction and demyelination in CPZ mice. The nuclear factor Nrf2 mainly modulates the endogenous cellular antioxidant response. Activation of Nrf2 suppresses oxidative stress and inflammatory responses in the CNS in MS. The Nrf2 agonist dimethyl fumarate has been approved for clinical treatment of MS. Astrocytes are the primary cell type that activates Nrf2 under pathological conditions in the CNS. Nrf2 activation is regulated by the IGF/PI3K/Nrf2 signaling pathway. Studies have found that IGF-1 knockout mice have reduced Nrf2 expression and increased oxidative stress and apoptosis. IGF-1 also reduces neuronal death by activating the PI3K/Nrf2 pathway. GK treatment upregulated Nrf2/HO-1 and downregulated p-NF-κB/p65 in astrocytes, inhibiting oxidative stress-related NO and iNOS in astrocytes. In addition, activation of Nrf2 after GK treatment modulated the IGF/PI3K signaling pathway to attenuate oligodendrocyte apoptosis in corpus callosum [165].

Bixin is isolated from the seeds of *Bixa orellana* and approved by FDA for use in the food and cosmetic industries, which can cross the blood–brain barrier and can prevent oxidative DNA damage and inhibit lipid peroxidation [166]. Bixin can significantly improve symptoms and inflammatory demyelination damage in EAE mice, and reduce the release of TNF-α, IL-6, IL-8, IL-17, and IFN-γ. The TXNIP/NLRP3 inflammasome is critical in the pathogenesis of CNS disease. The TXNIP/NLRP3 inflammasome is highly expressed in the spinal cord of EAE mice and worsens demyelination. Oxidative stress is one of the major causes of CNS dysfunction in MS, and CD4+ T cells produce large amounts of ROS, which are major mediators of oxidative stress and initiators of the TXNIP/NLRP3 inflammasome. Bixin can clean excess ROS and reduce the number of Th1/Th17 cells, which was achieved by activating Nrf2 signaling pathway and suppressing the activation of the TXNIP/NLRP3 inflammasome [167].

Olibanum is a hard gel-like resin exuded from *Boszvellia carterii* Birdw. and *Boswellia bhaw-dajiana* Birdw. Olibanum contains a series of complex components, of which the major active ingredient is 11-keto-beta-boswellic acid (AKBA), a resinous pentacyclic triterpenoid. AKBA has various physiological effects, including anti-infective, antitumor and antioxidant effects [168]. Treatment with AKBA alleviated demyelination and clinical symptoms in EAE mice. There is evidence that IL-6 amplifies neuroinflammation by activating immune cells of resident and peripheral origin. In MS/EAE, high IL-6 expression in the CNS was associated with elevated p-NF-κB levels. In EAE mice, oxidative damage markers such as lipid peroxides were elevated. iNOS participates in the MS neuroinflammation process by producing oxidants, and the production of iNOS is regulated by the transcription factor NF-κB. Activation of NF-κB leads to upregulation of IL-6 and iNOS-mediated oxidative stress, which is accompanied by downregulation of Nrf2 in a mouse model of EAE. AKBA treatment inhibited NF-κB signal transduction and activated the Nrf pathway, thus correcting the overexpression of IL-6 and iNOS, and alleviating the immune-inflammatory response in EAE. Therefore, AKBA may have a good therapeutic effect on relapsing-remitting MS [169].

Ginsenosides are triterpene saponins (terpene glycosides), of which ginsenoside Rg_3_ is a natural steroid saponin found in high contents in Korean red ginseng. Compared with other ginsenosides, ginsenoside Rg_3_ has a wide range of pharmacological activities, and it shows neuroprotective effects in various CNS disease models [170]. Ginsenoside Rg_3_ decreased demyelination and increased the integrity of the blood–brain barrier in EAE mice with CNS. COX-2, iNOS, IL-1β, IL-6 and TNF-α have downregulated after ginsenoside Rg_3_ treatment, while Arg-1 and IL-10 levels were elevated. Activated NOX2 and NOX4 can produce ROS released into the intracellular or extracellular space, aggravate the oxidative damage of oligodendrocytes, and lead to progressive demyelination. Ginsenoside Rg_3_ could also inhibit the expression of oxidative stress markers, including mtROS, NADPH, NOX2, NOX4 and 4-Hydroxynonenal (4-HNE) in the spinal cord of EAE mice [171].

Astragaloside (AST) is a tetracyclic triterpenoid saponin from Astragali Radix that has been shown to have good therapeutic effects in CNS demyelinating disorders [172]. AST administration significantly inhibited severity and blood–brain barrier leakage in EAE mice, reduced inflammatory cell infiltration and ROS production in CNS, upregulated SOD activity, and reduced neuroinflammation by inhibiting iNOS. In addition, different subtypes of T cells affect the occurrence and development of MS. Transcription factors T-bet, RORγt and Foxp3 contribute to the differentiation of CD4 naive T cells into distinct subpopulations. In the presence of IL-12 and T-bet expression, naive T cells can be induced to differentiate into Th1 cells; when TGF-β, IL-6 and retinoic acid receptor related to orphan receptor gamma-t (RORγt) are present together, Th17 cells will be generated. Foxp3 is essential for the development and function of Tregs. AST increased the mRNA expression of transcription factors T-bet and Foxp3 in the peripheral immune system of EAE mice but decreased the expression of ROR*γ*t to regulate T cell differentiation [173].

### 4.3. Phenylpropanoids

Nordihydroguaiaretic acid (NDGA), a phenolic lignan originally isolated from the leaves of *Larrea tridentata*, acts as one of the inhibitors of LOX and can suppress the inflammatory reaction. NDGA exerts neuroprotective effects by scavenging ROS and activating HO-1 in EAE mice. The p38MAPK signaling pathway regulates IL-17 synthesis in CD4+ T cells. SGK1 is one of the substrates of p38MAPK, and oxidative stress stimulates SGK expression through a p38/MAPK-dependent pathway. There is a strong correlation between oxidative stress and the p38MAPK-SGK1 pathway in T cell-mediated autoimmune inflammation. The p38MAPK-SGK1 pathway is the confluence of CNS oxidative stress and Th17 inflammatory responses. Oxidative stress activates the p38MAPK-SGK1 pathway and enhances the Th17 phenotype, thereby inducing pathological damage and neurological dysfunction. Oxidative stress p38MAPK-SGK1 pathway may be a central link in EAE, and MS. NDGA attenuates immune inflammation and oxidative stress in EAE mice by inhibiting p38MAPK-SGK1 pathway. Behavioral and pathological myelin damage is improved in NDGA-treated EAE mice, IL-17 and MDA are decreased in the spinal cord, and the antioxidant HO-1 is upregulated [174].

Caffeic acid phenethyl ester (CAPE) is a natural active compound derived from propolis, a catechol-containing phenylpropanoid derivative with multiple biological activities [175]. CAPE has significant advantages in antibacterial, antioxidant, anti-inflammatory and anti-cytotoxic properties. CAPE treatment significantly improved the clinical symptoms in EAE rats. In addition, CAPE may reduce myelin damage and destruction by inhibiting NF-*κ*B activation-mediated inflammatory responses, reducing ROS production, and exerting its antioxidant effects by downregulating iNOS, MDA, MPO, NO levels and increasing GPx, SOD activity in CNS tissues [176]. It has been found that ROS production is critical for NF-κB activation. However, CAPE inhibits NF-κB activation by inhibiting the interaction of NF-κB protein with DNA, not by maintaining IκB-α expression. CAPE reduces NF-κB activation by reducing the release of reactive oxygen species intermediates in the CNS of EAE rats. Xanthine oxidase (XO) is a physiological source of superoxide anion in eukaryotic cells. Tissue XO activity was significantly increased in the EAE group, while CAPE treatment reduced its expression, alleviating the overgenerated free radicals in the spinal cord of EAE rats.

P-Coumaric acid (p-CA), also known as 4-hydroxycinnamic acid, is a derivative of cinnamic acid and is present in free form in many plants [177]. Its activity in reducing oxidative stress and inflammatory responses have been demonstrated in various animal models, and it has been extensively studied due to its beneficial effects on CNS disease. Matrix metalloproteinase 9 (MMP-9) is secreted by activated lymphocytes, endothelial cells, and leads to disruption of the blood–brain barrier. It is essential to promote T cell migration to the CNS and induce demyelination during MS. Studies have shown that the expression of MMP-9 is regulated by oxidative stress level. In a CPZ-induced MS mouse model, treatment with p-CA by gavage significantly inhibited high expression of MMP-9 in the brain, protected integrity of the blood–brain barrier, and reduced the levels of the free radical indicator DPPH [178].

Magnolol (MAG), a phenylpropanoid derived from *Magnolia officinalis*, is a hydroxylated biphenyl natural compound. MAG can significantly reverse the clinical symptoms, pain parameters and CNS pathological changes of EAE and reduce the level of cytokines in a dose-dependent manner. Low expression of Nrf2 is associated with oxidative stress-mediated oligodendrocyte apoptosis in EAE. In vitro and in vivo studies have shown that Nrf2 attenuates CNS tissue damage. In contrast, failure of Nrf2 signaling renders the CNS more vulnerable and leads to excessive protein accumulation and neurodegeneration. In MS/EAE, Caspase-3 is a significant player in neuronal apoptosis, and inhibition of Caspase-3 activation in brain tissue can effectively protect neurons and oligodendrocytes. MAG treatment significantly inhibited oxidative stress by downregulating the activities of MDA, NO and MPO, and increased the levels of antioxidants, such as GSH, GST, cat and SOD. In addition, MAG significantly enhanced the antioxidant defense system by increasing the expression level of Nrf2 while decreasing the expression of iNOS and cleaved-caspase-3 in the brain to protect myelin integrity [179].

Daphnetin (DAP) is a 7, 8-dihydroxycoumarin extracted from *Daphne odora*. It has been shown in several preclinical animal studies and cellular experiments to be a potent neuroprotectant that effectively blocks neuronal damage in the CNS [180]. DAP-treated EAE mice have decreased brain levels of cytokines, including IL-17, INF-γ, IL-6, IL-12a and IL-23a. Overactivated Th1 and Th17 cells and inflammatory responses may induce oxidative stress in the progression of MS/EAE. HO-1 is a stress-responsive enzyme with potent antioxidant activity, and up-regulation of HO-1 expression inhibits inflammation-induced oxidative stress. Studies have shown that knockdown of HO-1 significantly increases the expression of IL-6, TNF-α, and IL-1β. These cytokines may be associated with the generation of Th1 and Th17 cells and the exacerbation of inflammatory pathology in EAE. DAP inhibited the production of IL-1β, IL-6 and TNF-α in LPS-stimulated BV2 cells. HO-1, a typical antioxidant and anti-inflammatory factor, was induced in large amounts in BV2 cells after daphnetin treatment. In addition, a significant elevation of HO-1 was observed in the brains of daphnetin-treated EAE mice, along with downregulation of MDA levels [181].

### 4.4. Alkaloids

Piperine, an alkaloid compound belonging to the amide group isolated from the fruit of black pepper (*Piper nigrum*), has important pharmacological activities, including in MS/EAE treatment. We all know that iNOS is one of the major oxidation-related enzymes in MS. It catabolizes arginine to generate NO in the environment of CNS injury, which affects the process of cytotoxicity, inflammation, and oxidative stress injury. The high expression of iNOS in the brain hinders the recovery of MS. Nrf2 regulates the expression of numerous antioxidant genes, including HO-1. HO-1 catalyzes the degradation of heme and produces carbon monoxide, ferrous iron, and biliverdin, thereby reducing oxidative damage. Administration of piperine attenuated neurological deficits, inhibited disease progression, reduced demyelination, inflammation, immune cell infiltration, microglia and astrocyte activation, and enhanced the expression of IL-10, Nrf2, HO-1, MBP and NeuN in EAE rats. Piperine also enhanced the total antioxidant capacity (FRAP) and reduced the levels of oxidative stress markers (MDA) in the CNS of EAE rats, while replenishing deficient BDNF and reducing neuronal apoptosis. Similarly, the results were consistent with the EAE model in LPC-induced demyelinated rats treated with piperine [182,183].

Matrine (MAT), a tetracyclo quinolizine alkaloid, is the main active ingredient extracted from the dry root of *Sophora flavescens* and used in a variety of CNS and peripheral inflammatory diseases [184]. Intraperitoneal MAT injection effectively inhibited EAE progression and significantly reduced oligodendrocyte apoptosis, microglial activation, and inflammatory factor secretion. The energy and redox state of mitochondria play a decisive role in cellular homeostasis. Mitochondrial dysfunction is associated with the degeneration of myelin sheaths and axons. ROS mainly originates from the electron leakage of mitochondrial complexes. During apoptosis, the abnormal structure and function of mitochondria lead to swelling of organelles, and then the cytoplasm releases Cyt-c and other apoptotic factors. Mitochondrial autophagy, a major pathway for maintaining mitochondrial function, has profound effects on the repair of damaged oligodendrocytes. MAT treatment also reduced the levels of Cyt-c and the oxidative stress marker MDA in CNS tissues. In contrast, autophagy-related proteins Beclin1, LC3 and GPx were upregulated after MAT treatment, thereby enhancing mitochondrial autophagy and alleviating the imbalance of oxidative/antioxidant systems induced by mitochondrial injury [185].

### 4.5. Quinones

Shikonin is a class of natural naphthoquinones extracted from the roots of *Lithospermum erythrorhizon*. Shikonin treatment of EAE mice significantly reduced the extent of corpus callosum demyelination. The level of ROS in MS increased, and the antioxidant defense system in vivo was damaged, which led to the enhanced blood–brain barrier permeability. Various inflammatory factors enter the brain and spinal cord in large quantities. Among them, TNF-α directly induces oligodendrocyte death and oligodendrocyte progenitor cell loss. IFN-γ can promote the inflammatory response of EAE and MS. The accumulation of these cytokines significantly increased the expression level of apoptosis-related proteins in CNS. Conversely, TGF-β is an anti-inflammatory cytokine, mainly produced by T cells, monocytes, astrocytes, and microglia, which can prevent autoimmune response and inflammatory damage. The genes expression of TNF-α, IFN-γ and Bax was enhanced and TGF-β and Bcl2 were reduced in the brain tissue of EAE mice, and shikonin treatment significantly reduced the expression levels of TNF-α, IFN-γ and Bax. In addition, the expression levels of TGF-β and Bcl2 and the activity of GPx1 were significantly increased after shikonin treatment. These results suggest that shikonin has good immunomodulatory and antioxidant effects in EAE and may contribute to the remission of EAE [186]. 

Thymoquinone (TQ), a benzoquinone, is a natural antioxidant isolated from the seeds of *Nigella sativa* and present in other plants [187]. Increased IL-17 and IL-17R have been reported in an EAE-induced demyelination mouse model, and treatment with TQ reduced this abnormal expression. In addition, TQ inhibited the development of acute and chronic recurrent EAE in mice, reduced the number of perivascular inflammatory cell infiltrates and increased GSH in the spinal cord, suggesting that TQ may reduce myelin damage in EAE mice by inhibiting oxidative stress [188,189].

### 4.6. Steroids

Withametelin (WMT), a natural sterol lactone derived from the leaves of *Datura stramonium*, has been shown to relieve depression and neuropathic pain significantly [190]. Dysfunction of the blood–brain barrier (BBB) plays a critical role in the pathogenesis of MS, with the migration of pro-inflammatory cells and toxic molecules to the brain through the damaged BBB, leading to demyelination and neuronal death. Fourier transform infrared spectroscopy revealed that EAE induced significant changes in myelin biomolecular composition, including protein oxidative damage, lipid peroxidation, increased nucleic acid/carbonyl content, and decreased lipid/protein content. It is well known that Nrf2/Keap-1-mediated oxidative stress and neuroinflammation contribute to neuronal degeneration in EAE models of MS. Oxidative stress in the brain, spinal cord, and optic nerve may cause permanent cellular damage due to the oxidation of cellular components. In MS, low levels of antioxidant enzymes and high levels of reactive oxygen species aggravate CNS damage. WMT treatment significantly attenuated EAE-induced weight loss, neuropathic pain, and motor dysfunction reduced elevated circulating leukocytes and blood–brain barrier disruption, and reversed histopathological changes in the brain, spinal cord, and optic nerve. WMT enhanced the antioxidant defense mechanism and decreased the expression of Keap-1 and iNOS by increasing the expression levels of Nrf2 and HO-1 in the CNS [191].

Guggulsterone (GST) is a natural phytosterol from the resin of *Commiphora mukul*, which is widely used in preclinical animal studies of CNS diseases such as ischemic stroke, dementia, depression, and autism [192]. PPAR-γ plays an important role in neuroinflammation, and up-regulation of PPAR-γ can reduce pathological expression in EAE models. Elevated PPAR-γ enhances remyelination in MS by suppressing T cells. It has been reported that PPAR-γ agonists reduce ROS production, protect mitochondria, and promote oligodendrocyte differentiation and maturation. Conversely, inhibition of JAK/STAT-mediated glial activation was neuroprotective, reduced interleukin and Th1 cell differentiation, and attenuated oxidative damage. GST improves behavioral deficits (spatial cognitive memory, grip and motor coordination) and increases the expression of the myelin marker MBP in EB demyelinated rats. GST also modulates neurotransmitter levels by increasing acetylcholine, dopamine, serotonin and decreasing glutamate. In addition, GST ameliorates inflammatory cytokines (TNF-α, IL-1β) and oxidative stress markers (AchE, SOD, CAT, MDA, GSH, NO) to prevent EB-induced apoptosis. These effects were associated with the dowregulation of JAK/STAT and upregulation of PPAR-γ signaling pathways by GST [193].

### 4.7. Other Compounds

Sulforaphane (SFN) is an organosulfur compound derived from cruciferous vegetables (e.g., cauliflower). SFN exerts neuroprotective effects through its antioxidant effect in CNS diseases such as Alzheimer’s disease, Parkinson’s disease, epilepsy, etc. It is reported that ROS reduces the integrity of BBB and facilitates the entry of peripheral immune cells into the CNS. Infiltrated leukocytes produce ROS and induce oligodendrocyte and axon damage. In addition, reactive microglia produce peroxynitrite, the main mediator of oxidative stress and neuronal excitotoxicity, thus driving the neurodegenerative process in MS. Nrf2 is a redox-sensitive transcription factor. Previous studies have shown that Nrf2/ARE transcription pathway is crucial for cell defense against oxidative damage. SFN treatment inhibits inflammatory infiltration, demyelination and upregulation of iNOS and NO in the spinal cord of EAE mice. Another study showed that SFN protected the blood–brain barrier in EAE mice and by upregulating Nrf2/ARE pathway to activate the antioxidant HO-1 and NADPH quinone oxidoreductase 1 (NQO1) expression levels. In addition, SFN treatment can inhibit Th17 response and enhance the release of IL-10. These results suggest that SFN inhibits the development of EAE in mice through its antioxidant and anti-autoimmune inflammatory activities [194,195].

3H-1,2-dithiole-3-thione (D3T) is a compound containing a five-membered cyclic sulfur structure extracted from cruciferous vegetables. D3T effectively induces activation of cellular antioxidant and anti-inflammatory defense systems and provides protection in a variety of disease models. In MS/EAE, antigen-presenting cells activate naive T cells and produce inflammatory cytokines to promote the differentiation of encephalitogenic CD4+ T cells. Dendritic Cells (DCs) have been shown to play a key role in promoting the development of pathogenic Th1/Th17 cells. The proinflammatory cytokines IL-12 and IL-23 produced by DCs have been shown to be critical for Th1 and Th17 differentiation, respectively. The administration of D3T after the onset of EAE effectively prevents disease progression. Pharmacological studies have shown that D3T inhibits dendritic cell activation, suppresses the differentiation of Th1 and Th17, and inhibits microglia activation and inflammatory cytokine expression. In vitro experiments revealed that D3T strongly induced Nrf2 and HO-1 expression and enhanced antioxidant activity in LPS-stimulated dendritic cells [196].

C-Phycocyanin (C-Pc) is a photosynthetic pigment isolated from *Spirulina platensis* with significant effects in regulating excessive oxidative stress, inflammatory damage and immune responses. There was a massive inflammatory infiltration consisting of lymphocytes and macrophages/activated microglia in animals with EAE. Microglia comprise about 10% of all brain cells and are the first line of CNS immune defense. Most studies have shown that activated microglia exacerbates MS/EAE pathogenesis by producing neurotoxic molecules, proinflammatory cytokines, and oxygen-free radicals. Furthermore, IL-17 and IL-6 are the primary effector cytokines in MS, and their knockout mice are protected from EAE. Therefore, it is necessary to intervene in immune cell-mediated inflammatory responses and oxidative damage. C-Pc can ameliorate clinical deterioration, reduce Inflammatory macrophage/microglia infiltration in spinal cord tissue and IL-6, IL-17 in the brain and serum, and regulates oxidative stress parameters (lower MDA, higher GSH) in the peripheral blood of EAE mice, thereby slowing oxidative damage and increasing myelin repair and regeneration in the CNS [197].

### 4.8. Plant Extracts

*Artemisia dracunculus* L. is a perennial herb of the Asteraceae family and is an important herb in traditional medicine in many countries. Modern pharmacological studies have demonstrated that it has inhibitory effects on inflammation and oxidative damage, and hepatoprotective and neuroprotective effects [198]. In MS/EAE, Th17 cells can produce IL-17 and IL-23. IL-17 can inhibit the differentiation and maturation of oligodendrocytes. Moreover, IL-17 can enhance the apoptosis-induced effect of TNF-α in oligodendrocytes, which is mainly related to mitochondrial dysfunction, ROS generation, and cell cycle arrest. Administration of *A. dracunculus* aqueous extract alleviated weight loss, inflammatory infiltration and demyelination of CNS in EAE mice. After treatment, serum levels of inflammatory cytokines, including IL-17 and IL-23, were reduced, while antioxidant levels (FRAP) were increased [199].

Olive leaves are widely used as a medicinal plant in the Mediterranean region. Some evidence suggests that it contains high levels of phenolics with antioxidant effects to prevent and treat neurodegenerative diseases. The reduction of SIRT1 exacerbates the progression of various neurodegenerative diseases, including MS. Inhibition of SIRT1 expression may contribute to microglial activation and neuroinflammation. SIRT1 has been shown to affect the redox properties of cells and reduce oxidative stress by regulating FOXO3a, resulting in increased activity of CAT and SOD. Based on these facts, SIRT1 is a promising target for MS therapy. Oral administration of olive leaf tea combined with olive leaf extract intraperitoneally in EAE mice attenuated the severity of MS and suppression of SIRT1, upregulated antioxidant enzymes (SOD1, SOD2 and GPx1) and M2 microglia, and inhibited M1 phenotype to maintain myelin integrity [200].

*Melilotus Officinalis* is an herb from traditional medicine, mainly distributed in East Asia, the Middle East and the eastern shores of Mediterranean, with good anti-inflammatory effects [201]. Disruption of Th1/Th2 balance and oxidative damage and apoptosis of oligodendrocytes play a key role in the pathogenesis of MS. IL-6, TNF-α, IL-12, and IFN-γ are secreted by Th1 cells, but Th2 cells release anti-inflammatory cytokines, such as IL-4, and IL-5, which are vital factors affecting axonal and myelin damage. Prophylactic administration of Melilotus Officinalis extracts attenuated clinical symptoms and pro-inflammatory factors such as IL-6, TNF-α and IFN-γ in the corpus callosum of EAE mice. This herbal extract also promoted the expression of anti-inflammatory cytokines and antioxidant enzymes (CAT, GPx1), thereby maintaining the structural integrity of myelin [202].

*Xanthoceras sorbifolia* Bunge has long been used in China as traditional folk medicine [203]. Its fruit shell extract (NE) contained high natural phenolic compounds and potent antioxidants. In vivo results showed that oral administration of NE effectively improved clinical disease severity and reduced CNS demyelination in EAE mice. The phosphorylation of STAT1 and STAT3 was highly expressed in CNS of EAE mice, which promoted the polarization of Th1/Th17 cells. NE inhibited Th1 and Th17 cell differentiation via modulating JAK/STAT signaling pathway and reduced the entry of brain inflammatory immune cells into the CNS. In addition, NE exhibited extremely strong antioxidant capacity in vitro and reduced the level of DPPH free radicals [204].

Saffron comes from the flowers of *Crocus sativus* L., a common medicinal plant whose neuroprotective effects in CNS disorders such as depression, anxiety, Alzheimer’s, Parkinson’s, and epilepsy have received much attention in recent years. In vivo studies showed that oral administration of saffron extracts significantly delayed the onset of EAE disease and attenuated clinical symptoms and CNS inflammatory cell infiltration in mice. Oxygen and nitrogen free radicals lead to lipid peroxidation in MS/EAE, exacerbating neuronal and oligodendrocyte damage. Improving antioxidant enzyme activity is beneficial in preventing free radical-mediated CNS inflammatory response and tissue damage. In addition, total antioxidant capacity (TAC) in the serum of EAE mice treated with saffron extracts was significantly increased [205].

*Moringa oleifera* is a tree that grows widely in many tropical and subtropical countries. The seeds, leaves, flowers and oil of *Moringa oleifera* are widely used in Southeast Asian traditional medicine. *Moringa oleifera* has good antioxidant, antidiabetic, antihyperlipidemic, and cardiovascular and neuroprotective effects. CPZ-induced demyelination resulted in memory impairment, increased cortical and hippocampal oxidative stress (inhibited CAT, SOD, increased NO release), and neuronal damage in rats. There is a marked intracellular accumulation of nitrotyrosine-positive protein aggregates in neurodegenerative diseases such as MS. However, administration of *Moringa oleifera* significantly reversed CPZ-induced neuropathological defects and nitrative stress, while enhancing the antioxidant capacity of rat brain [206]. Olive oil is extracted from the olive fruit and more than 200 compounds, including sterols, carotenoids, triterpene alcohols, and phenols, have been detected in olive oil. The microbiota products (LPS and LBP) in EAE were positively correlated with oxidative stress. LPS and LBP induce activation of the peripheral immune system and increase the permeability of the blood–brain barrier, leading to the persistence of oxidative damage to the CNS. EAE rats treated with olive oil by gavage for 51 days showed a reduction in bacterial LPS and LPS binding protein (LBP) in the brain, spinal cord and blood. LPO and NO, indicators of lipid and protein oxidation, were downregulated and the activity of antioxidant enzymes GSH and GPx was increased, thus slowing down oxidative damage to CNS myelin [207].

Copaiba oil (COP), an oleoresin extracted from *genus Copaifera* L., is an important phytomedicine in South American traditional medicine with significant bactericidal and anti-inflammatory effects. H_2_O_2_ is widely generated in the CNS of MS/EAE, and prolonged exposure to high concentrations of H_2_O_2_ can lead to irreversible cell damage. Similar to H_2_O_2_, NO is responsible for myelin damage in MS. NO is neurotoxic and is abundantly produced by macrophages and other immune cells. In vitro experiments confirmed that EAE mouse splenocytes were overproduced with oxidative mediators (H_2_O_2_, NO) and pro-inflammatory factors (IFN-*γ*, TNF-*α* and IL-17) under the stimulation of MOG_35–55_ and ConA, and oxidative stress and inflammatory responses were significantly inhibited after co-culture with COP for 24 and 48 h [208].

*Sesamum indicum*, traditional health food in many Asian countries, contains many fat-soluble antioxidants. Daily intraperitoneal administration of sesame oil to EAE mice after immunization effectively delayed the onset of EAE and reduced clinical symptoms. Sesame oil can significantly improve the total antioxidant capacity of serum and inhibit the production of NO. Typical brain inflammatory cell infiltration was observed in EAE mice compared to sesame oil-treated mice. This result suggests that sesame oil effectively prevents disease progression in EAE, which may be related to the inhibition of oxidative stress [209].

*Oenothera biennis* L. belongs to the Onagraceae family and has long been used in folk medicine as a good natural anti-inflammatory and antioxidant agent [210]. *Hypericum perforatum* belongs to the Hypericaceae family and its beneficial effects have been demonstrated in treating depression, tumors, and bacterial infections [211]. Recently, the therapeutic effects of *O. biennis* and *H. perforatum* on EAE have been reported. Oxygen and nitrogen free radicals produced by macrophages and other immune cells are involved in demyelination and axonal damage in MS. Antioxidants can prevent brain tissue damage caused by free radicals. MS/EAE brain and spinal cord tissue increased TOS and OSI levels and decreased TAS expression. The result showed that extracts of *O. biennis* and *H. perforatum* decreased the levels of TOS and OSI, increased TAS levels and reduced clinical symptoms and myelin damage in the brains of EAE mice. EAE mice were also observed to have amyloid deposition in the vessel wall, neuronal cytoplasm and cell interstitial spaces. These abnormal expressions were significantly eliminated in *O. biennis* and *H. perforatum* extract-treated groups [212].

## 5. Conclusions

Autoimmunity-induced oxidative stress is particularly crucial for the onset and progression of MS because it is involved in the activation and recruitment of immune cells, disruption of the blood–brain barrier, damage to neurons and oligodendrocytes, and other key aspects of MS. Oxidative stress and inflammatory damage to the CNS form a vicious cycle. In addition, the weakened antioxidant defense system in patients and animals with MS can lead to increased oxidative stress. ROS/RNS in the CNS of patients with MS is initially derived from activated microglia and reactive astrocytes. ROS/RNS attack neurons and oligodendrocytes, inducing oxidative damage to lipids, proteins and DNA, resulting in damaged cell mitochondria. The ensuing collapse of the damaged cellular mitochondrial membrane potential and mitochondrial dysfunction further leads to more free radical production, exacerbating the cytokine storm-mediated inflammatory responses and demyelination in the brain, spinal cord and optic nerves.

To date, several plants and their active ingredients have been studied to prevent and treat neurodegenerative pathologies such as MS. Most of them have focused on the immunomodulatory, antioxidant, anti-inflammatory and anti-apoptotic effects of the agents. In particular, natural antioxidants rich in compounds such as phenolics, terpenoids and alkaloids have positive effects in reducing myelin damage in MS. Overproduction and inefficient scavenging of free radicals leads to oxidative stress in MS. Enhancing the antioxidant system and inhibiting oxidative responses are promising targets for MS therapy. The majority of the medicinal plants and active ingredients summarized in this review increase the level of antioxidant enzymes in the CNS and reduce the production of ROS/RNS to exert anti-demyelinating neuroprotective effects. In the EAE model, many natural antioxidants can protect the integrity of the blood–brain barrier, inhibit the differentiation of Th1/Th17 cells and the activation of macrophages, reduce the production of oxygen/nitrogen free radicals and pro-inflammatory cytokines, increase anti-inflammatory factors, and regulate the phenotype of microglia to maintain the immune balance in the CNS and mitigate axon and myelin damage. Besides, when natural products with antioxidant properties treat the animal demyelinating models induced by toxicity (such as CPZ, LPC and EB), they can effectively improve the activity of endogenous antioxidant enzymes, reduce mitochondrial dysfunction, prevent the excessive production of ROS induced by mitochondrial electron leakage and membrane potential collapse, to reduce the apoptosis of oligodendrocytes and neurons and reduce the inflammatory level of brain tissue. The antioxidant effects of these natural products in treating MS are mostly related to the regulation of Nrf2/ARE, JAK/STAT, PPARγ, SIRT1 and p38MAPK-SGK1 signaling pathways. However, the efficacy and risks of using natural antioxidants against MS in humans have not been fully elucidated due to the lack of evidence from large samples collected for randomised controlled clinical trials. 

In conclusion, natural products with antioxidant properties have significant potential in the treatment of MS; however, several challenges persist in their use. Therefore, more clinical studies are required to determine whether these natural antioxidants have better efficacy in treating MS in humans, either alone or in combination with other drugs. This review brings potential natural antioxidants against MS from animal experiments (not fully accepted from the perspective of clinical practice) with very careful offer of the results to neurologists treating real patients with MS.

## Figures and Tables

**Table 1:** The anti-oxidative stress properties of natural products used in animal models of MS.

Compound	Experimental Model	Treatment Time Point and Path	Dosage	Antioxidative Stress Effect	Major Results
Anthocyanins	EB (male Wistar rats)	Starting from EB injection day (oral)	30, 100 mg/kg for 7 days	↑ HNE-His, MDA, NOX ↑ NPSH, GSH, SOD	↓ IL-6, IL-1β, TNF-α, IFN-γ. ↑ IL-10. ↑ Na^+^, K^+^-ATPase, Ca2+-ATPase.
Hesperidin	EAE (female C57BL/6J mice)	After 14th day induction of EAE (i.p. injection)	100 mg/kg/d for 7 days	↓ MDA. ↑ GPx, SOD, CAT, GSH	↓ Caspase-3, IL-17, TNF-α and IL-1β. ↓ inflammatory infiltration.
Licochalcone A	EAE (female C57BL/6 mice)	Starting at 10th days post-immunization (oral)	15, 30 mg/kg/d for 10 days	↓ H2O2, NO	↓ TNF-α, IFN-γ and IL-17. ↓ Th1 and Th17 cells.
Flavocoxid	EAE (female C57BL/6 mice)	Prevention protocol: Starting from immunization day therapeutic treatment protocol: from day 14 post immunization (i.p. injection)	100 mg/kg/d every other day Prevention protocol: for 28 days. therapeutic treatment protocol: for 19 days.	↓ iNOS, COX2, 5-LOX.	↓ IL-6, IL-12, IL-23, IL-17, IFN-γ. ↓ Th1/Th17 cells. ↑ Arg1, Ym1, CD206, TGF-β, IL-10.
Luteolin	EAE (female Wistar rats)	Starting at 10th day post-immunization (i.p. injection)	10 mg/kg/d for 33 day	↑ TAC	↑ CNTF, cAMP. ↓ c-caspase-3, NF-κB, MIP-1α.
Epimedium flavonoids	EAE (Female Lewis rats)	Starting from immunization day (oral)	20, 60 mg/kg/d for 15 days	↓ NO, iNOS	↓ IL-1β, TNF-α, IκB-α, Iba1, CD3, GFAP. ↑ CNPase, NGF.
Kolaviron	CPZ (male Wistar rats)	Starting from cuprizone diet fed (oral)	200 mg/kg/d both cuprizone and kolaviron for 42 days	↓ MDA. ↑ GPx, SOD	↑ the neuronal integrity
Quercetin	EB (male Wistar rats)	Starting from EB injection day (i.p. injection)	50 mg/kg/d for 22 days	↓ MDA. ↑ CAT, SOD.	↓ AChE. ↓ Na^+^, K^+^-ATPase
Phloretin	EAE (C57BL/6J mice)	Starting after 6 days of immunization or after disease onset (i.p. injection)	50 mg/kg/d for 10 days or 20 days	↓ ROS, NO, NOS2, ↑ Nrf2, NQO1	↓ MHC-II, CD86, TNF-α, IL-6, CCL4, CCL5, CXCL2. ↑ IL-4, CNTF, IGF-1, p-AMK, P62, LC3-II.
Curcumin	EAE (female C57BL/6 mice)	Starting from immunization day (i.p. injection)	20 mg/kg/d for 21 days	↑ GPx, SOD.	↓ IL-6, IL-17, TNF-α, IFN-γ. ↑ TGF-β.
	EAE (female Lewis rats)	Starting at 12th day post-immunization	12.5 mg/kg/d for 18 days	↓ iNOS, ↑ HO-1, Nrf2	↓ IL-1, IL-17. ↑ IL-4, IL-10, TGF-β, BDNF, NGF, MBP, Nestin.
Resveratrol	EAE (female C57BL/6 mice)	Starting from immunization day (i.p. injection)	10, 25, 50 mg/kg/d for 20 days	↓ iNOS, NOX2, NOX4. ↓ NADPH activity	↑ Arg1 and IL-10 ↑ ZO-1, Occludin, claudin-5, ICAM-1, VCAM-1. ↑ the BBB integrity
	CPZ (male C57BL/6 mice)	cuprizone diet for 7 days, followed by 3 weeks on 0.2 % cuprizone diet plus resveratrol (oral)	250 mg/kg/d for 21 days	↓ LPO. ↑ GSH, SOD, cytochrome oxidase	↓ Rel-A, pIκB-α, TNF-α. ↑ MBP, CNP, Olig1.
Acteoside	EAE (female C57BL/6N mice)	Prevention protocol: Starting from day 2 post immunization therapeutic treatment protocol: from day 11 post immunization (oral)	Prevention protocol: 30 mg/kg/d for 29 days therapeutic treatment protocol: 5, 10 and 30 mg/kg/d for 19 days	↓ ONOO-, iNOS, NADPH oxidases.	↓ LC3-II to LC3-I in mitochondrial fraction. ↓ the translocation of Drp1 to the mitochondria. ↓ neuronal apoptotic.
Oleacein	EAE (female C57BL/6J mice)	Starting from immunization day (i.p. injection)	10 mg/kg/d for 24 days	↓ O2^−^, MDA, AOPP, ROS, iNOS, COX2. ↑ FRAP, Sestrin-3	↓ TNFα, IL-13, IL-33, IL-1β, MCP-1. ↑ IL-10. ↑ the BBB integrity. ↓ inflammatory infiltration.
Ellagic acid	CPZ (male C57BL/6 mice)	Starting from cuprizone diet fed (oral)	5, 50, 100 mg/kg/d coadministration of CPZ and Ellagic acid for 6 weeks	↓ MDA, ROS. ↑ CAT, SOD.	↑ the integrity of myelin in spinal cord and sciatica.
Arbutin	LPC (male Wistar rats)	Starting from LPC injection day (i.p. injection)	50 mg/kg/d for 14 days	↑ Nrf2, HO-1.	↓ IL-1β, IL-17, TNF-α, GFAP. ↑ IL-10, MBP, Olig2.
Paeonol	CPZ (male C57BL/6 mice)	Started 1 week after beginning cuprizone challenge till the end of week 6 post-cuprizone (oral)	25 mg, 100 mg/kg/d for 42 days	↓ MDA, ROS, MPO. ↑ CAT, SOD, GSH.	↓ NF-κB, TNF-α. ↑ MBP.
18β-glycyrrhetinic acid	EAE (female C57BL/6 mice)	14 days after induction of EAE (i.p. injection)	100 mg/kg/d for 7 days	↓ MDA. ↑ GPx, SOD, CAT, GSH	↓ Caspase-3, IL-17, TNF-α, IL-1β.
β-Caryophyllene	EAE (female C57BL/6 mice)	Starting at 10th day post-immunization (oral)	25, 50mg/kg/d for 9 days	↓ H2O2, NO	↓ TNF-α, IFN-γ, IL-17
Ginkgolide K	CPZ (male C57BL/6 mice)	Starting from 5th weeks until the end of 6th weeks (oral)	20 mg/kg/d for 14 days	↑ Nrf2, HO-1. ↓ NO, iNOS.	↓ IL-1β, IL-6, TNF-α. ↓ p-NF-kB/p65, caspase-3, TUNEL. ↑ IGF/PI3K.
Bixin	EAE (female C57BL/6 mice)	Starting at 12th days post-immunization (i.p. injection)	50, 100, 200 mg/kg/d for 18 days	↓ ROS, Nrf2, TXNIP, NLRP3	↓ TNF-α, IL-6, IL-8, IL-17, and IFN-γ. ↑ IL-10. ↓ Th1 and Th17 cells.
Acetyl-11-keto-β-boswellic acid	EAE (female SJL/J mice)	starting at day 11 after immunization (oral)	20 mg/kg/d for 30 days	↑ Nrf2, HO-1, TAC ↓ iNOS, lipid peroxides	↓ p-NF-κB, NF-κB, IL-6
Ginsenoside-Rg3	EAE (female C57BL/6N mice)	Starting from 7 days before immunization (oral)	37.5, 75, 150mg/kg/d for 28 day	↓ MitoSOX, 4-HNE, NOX2, NOX4, NADPH activity, COX2, iNOS	↑ occludens-1, claudin-3, and claudin-5. ↓ TNF-α, IL-6, IL-1β.
Astragaloside	EAE (female C57BL/6 mice)	Starting from immunization day (i.p. injection)	10, 25, 50 mg/kg/d for 15 days	↓ ROS, MDA, iNOS ↑ GPx, SOD	↓ p53, p-tau ↑ Bcl-2/Bax ratio. ↑ the BBB integrity.
Nordihydroguaiaretic acid	EAE (female C57BL/6 mice)	Starting from immunization day (i.p. injection)	10 mg/kg/d for 30 days	↓ MDA. ↑ HO-1	↓ IL-17 ↓ p38MAPK, SGK1. ↓ inflammatory infiltration.
Caffeic acid phenethyl ester	EAE (female Wistar rats)	Starting from the first day of immunization (i.p. injection)	25 μmol/kg/d for 14 days	↑ GPx, ADA, SOD. ↓ MDA, NO, XO.	↓ inflammatory infiltration.
P-Coumaric Acid	CPZ (female and male C57BL/6 mice)	Starting from 7th weeks until the end of 12th weeks (oral)	21.6 ppm/mouse/d for 42 days	↓ free radical (DPPH)	↓ MMP-9
Magnolol	EAE (female Swiss mice)	Starting from immunization day (oral)	0.1, 1, 10 mg/kg/d for 21 days	↓ MDA, MPO, NO, iNOS. ↑ GSH, GST, SOD, Nrf2.	↓ CD8+ T cell. ↓ c-caspase-3.
Daphnetin	EAE (female C57BL/6 mice)	Starting from immunization day (i.p. injection)	8 mg/kg/d for 21 days	↓MDA, HO-1	↓ IFN-γ, IL-17, IL-1β, IL-6, TNF-α. ↑ IL-10. inflammatory infiltration.
Piperine	EAE (female Lewis rats)	Starting at 8th day post-immunization (i.p. injection)	5 mg/kg/d for 22 days	↓ iNOS, MDA. ↑ Nrf2, HO-1 FRAP.	↓ TNF-, IL-1β, caspase-3. ↑ IL-10, BDNF, MBP.
	LPC (Male Wistar rats)	Starting at 3 days post LPC injection (i.p. injection)	5, 10, 20 mg/kg/d for 10 days	↑ Nrf2, HO1, FRAP. ↓ iNOS.	↓ TNF-α, IL-1β, NF-κB, Foxp3. ↑ IL-10, BDNF, MBP.
Matrine	EAE (Female Wistar rats)	starting from day 11 post immunization (i.p. injection)	250 mg/kg/d for 7 days	↓ MDA. ↑ GPx	↓ caspase-3, α-B-crystallin, Cyt. ↑ Beclin1, LC3.
Shikonin	EAE (female C57BL/6 mice)	After EAE was induced (i.p. injection)	20 mg/kg/d for 21 days	↑ GPx1	↓ TNF-α, IFN-γ and Bax. ↑ TGF-β and Bcl2.
Thymoquinone	EAE (female Lewis rats)	at days 1–5 post immunization or at day 12–17 post immunization (i.v. injection)	1 mg/kg for 5 days or 6 days	↑ GSH	↓ IL-7, IL-7R ↓ inflammatory infiltration.
Withametelin	EAE (Female Swiss mice)	starting at day 9 through day 25 (i.p. injection)	10, 100, and 1000 μg/kg/d for 17 days	↓ NO, MPO, iNOS ↑ GSH, SOD, Nrf2, HO-1	↓ TLR4, NF-κB, AP-1. ↑ IκB-α, Bcl-2.
Guggulsterone	EB (Wistar rats)	Starting at 8th day post EB injection (oral)	30, 60 mg/kg/d for 28 days	↓ AchE, MDA, NO. ↑ SOD, CAT, GSH.	↓ IL-1β, TNF-α. ↓ caspase-3, Bax, STAT3. ↑ Bcl2, PPAR-ϒ, MBP.
Sulforaphane	EAE (female C57BL/6N mice)	14 days before EAE induction (oral)	50 mg/kg/d for 28 days	↓ iNOS	↓ CD4, CD68, GFAP ↓ inflammatory infiltration.
	EAE (female C57BL/6 mice)	Starting from immunization day (i.p. injection)	50 mg/kg every other day up to 22 days	↓ HO-1, NQO1 ↑ Nrf2	↑ Occludin, claudin-5. ↑IL-10. ↓ MMP9, Th17 cells.
3H-1,2-dithiole-3-thione	EAE (female C57BL/6N mice)	starting at day 1 post immunization (i.p. injection)	10 mg/kg/d for 30 days	↑ Nrf2, NQO1, GCLC, HO-1. ↓ iNOS	↓ IL-23. ↓ Th1 and Th17 differentiation.
C-Phycocyanin	EAE (female C57BL/6 mice)	Treatments started at disease onset (i.p. injection)	2, 4, 8 mg/kg/d for 15 days	↓ MDA, peroxidation potential, CAT/SOD ratio. ↑ GSH.	↓ CD3, Mac-3 ↓ IL-17, IL-6, Foxp3 ↓ inflammatory infiltration
Artemisia dracunculus L.	EAE (female C57BL/6 mice)	Starting at 11th day post immunization (oral)	500 mg/kg/d for 23 days	↑ TAC (FRAP)	↓ IL-17, IL-23. ↑ TGF-β. ↓ inflammatory infiltration
Olive leaf	EAE (female C57BL/6 mice)	starting from the first day after EAE induction. (olive leaf tea, oral) starting from the 8th day after EAE induction (olive leaf extract, i.p. injection)	Olive leaf tea: ad libitum for 20 or 30 days Olive leaf extract: 1024 mg/kg/d for 10 days	↓ MDA ↑ SOD1, SOD2, GPx1	↑ SIRT. ↓ M1 microglia. ↑ M2 microglia.
Melilotus officinalis	EAE (female C57BL/6 mice)	starting from first day post-immunization (i.p. injection)	10 mg/kg/d for 21 days	↑ CAT, GPx1	↓ IL-6, TNF-α, IFN-γ, IL-17. ↑ TGF-β, IL-5.
Nutshell of Xanthoceras sorbifolia	EAE (female C57BL/6 mice)	Starting from 5 days before immunization (oral)	50, 100, 150 mg/kg/d for 35 days	↓ free radical (DPPH)	↓ Th1, Th17 cells. ↓ p-STAT1, p-STAT3, p-STAT4. ↓ IL-17, IL-1α, IL-1β, TNF-α, CCL1, CCL2, CXCL1, CXCL10, CXCL11.
Crocus sativus L.	EAE (male C57BL/6 mice)	Starting from immunization day (oral)	500 mg/kg/d for 21 days	↑ TAC (FRAP). ↓ NO	↓ inflammatory infiltration
Moringa oleifera.	CPZ (Wistar rats)	Starting from cuprizone diet fed (oral)	1.875 mg/mL/mouse/d for 35 days	↓ NO ↑ CAT, SOD	↑ neuronal integrity.
Olive oil	EAE (Dark Agouti rats)	starting at 11th day post immunization (oral)	representing 10% of calorie intake in the total standard diet for 54 days	↑ GPx. ↓ LPO, NO.	↓ NF-κBp65, TNF-α. ↓ LPS, LBP.
Copaiba oil	EAE (female C57BL/6 mice)	Splenocytes were obtained from EAE mice at day 20 post immunization (in vitro)	100, 50 and 25 µg/mL for 24 h	↓ H2O2, NO.	↓ TNF-α, INF-γ, IL-17
Sesame oil	EAE (male C57BL/6 mice)	Starting from day 3 before the immunization (oral)	4 mL/kg/d for 28 days	↓ NO ↑ TAC (FRAP).	↓ inflammatory infiltration.
Hypericum perforatum L. extract	EAE (C57BL/6 mice)	Starting from immunization day (oral)	18−21 g/kg/d for 42 days	↑ TSA. ↓ TOS, OSI.	↑ MOG, MBP. ↓ inflammatory infiltration.
Oenothera biennis L. extract	EAE (C57BL/6 mice)	Starting from immunization day (oral)	18−21 g/kg/d for 42 days	↑ TSA. ↓ TOS, OSI.	↑ MOG, MBP. ↓ inflammatory infiltration.

**Note:** “↑” represents upregulation and “↓” represents downregulation in the table.
